# Effect of Stress Ratio and Loading Frequency on the Corrosion Fatigue Behavior of Smooth Steel Wire in Different Solutions

**DOI:** 10.3390/ma9090750

**Published:** 2016-09-01

**Authors:** Songquan Wang, Dekun Zhang, Ningning Hu, Jialu Zhang

**Affiliations:** 1 School of Mechatronic Engineering, Jiangsu Normal University, Xuzhou 221116, China; huningning@cumt.edu.cn (N.H.); cumtzhangjialu@163.com (J.Z.); 2 School of Materials Science and Engineering, China University of Mining and Technology, Xuzhou 221116, China; dkzhang@cumt.edu.cn

**Keywords:** steel wire, corrosion fatigue, stress ratio, loading frequency

## Abstract

In this work, the effects of loading condition and corrosion solution on the corrosion fatigue behavior of smooth steel wire were discussed. The results of polarization curves and weight loss curves showed that the corrosion of steel wire in acid solution was more severe than that in neutral and alkaline solutions. With the extension of immersion time in acid solution, the cathodic reaction of steel wire gradually changed from the reduction of hydrogen ion to the reduction of oxygen, but was always the reduction of hydrogen ion in neutral and alkaline solutions. The corrosion kinetic parameters and equivalent circuits of steel wires were also obtained by simulating the Nyquist diagrams. In corrosion fatigue test, the effect of stress ratio and loading frequency on the crack initiation mechanism was emphasized. The strong corrosivity of acid solution could accelerate the nucleation of crack tip. The initiation mechanism of crack under different conditions was summarized according to the side and fracture surface morphologies. For the crack initiation mechanism of anodic dissolution, the stronger the corrosivity of solution was, the more easily the fatigue crack source formed, while, for the crack initiation mechanism of deformation activation, the lower stress ratio and higher frequency would accelerate the generation of corrosion fatigue crack source.

## 1. Introduction

Mine steel wire rope is an important part of the mine hoist transport system, which connects the hoisting container and the hoist. The lifespan of steel wire rope directly affects the safe operation and normal production of a coalmine [1,2]. The damage of mine steel wire rope occurs easily because of the bad working environment. A major accident will be caused by the fracture of mine steel wire rope. Mine steel wire rope is composed of cold drawn steel wires. Therefore, the failure analysis of cold drawn steel wire is always a hot issue. The corrosion, fatigue and wear are the three main failure forms [3], while the fatigue failure analysis of steel wire is the most widely studied. 

For example, in Beretta’s study [4], a model for the fatigue strength prediction of rope wires was presented and discussed. Within this method, the fatigue process of wires was described in terms of propagation of the surface defects caused by cold drawing. Verpoest [5] also thought that pearlitic steel wire propagated from pre-existing surface defects, which could be treated as crack. In Llorca’s study [6], the effect of stress ratio on fatigue threshold in cold drawn eutectoid steel wires has been experimentally measured. The practice shows that the working condition of mine steel wire rope has great influence on its life; for example, the high humidity and water spray can cause the corrosion on the surface of steel wire. Corrosion fatigue will occur easily under the common influence of complex external stresses, which will reduce the service life of steel wire. There are many factors affecting the corrosion fatigue life of metallic materials, such as the mechanical properties [7,8], the material properties [9,10,11], the environmental factor [12,13,14,15,16,17], and the stress states [18,19,20,21]. Among them, the corrosion environment can determine the corrosion reaction kinetics characteristic. As shown in Lin’s study [12], the high-cycle fatigue resistance of austempered ductile iron was dramatically reduced by the given aqueous media, in particular, to a greater extent with a decrease in pH value. In addition, the stress ratio [18,19,20] and loading frequency [20,21] are the two main stress states that affect the corrosion fatigue life of metal materials. As shown in Han’s study [19], the increase of crack growth rate would be caused by both decreasing frequency and raising stress ratio.

However, there are still many problems on the specific rules and related physical mechanisms about the effects of stress ratio and loading frequency on corrosion fatigue life of materials. As indicated above, a large number of researches were mainly focused on the influence of stress ratio and loading frequency on the corrosion fatigue crack growth rate of the specimens with pre-crack. The study of the effect of stress ratio and loading frequency on the crack initiation mechanism is relatively lacking, and, especially for smooth thin steel wire specimens, such research is particularly necessary. 

In order to determine the influence of various factors on the service life of steel wire, the kinetic parameters of corrosion process in different solutions were studied firstly to determine the effect of different solutions on the corrosion mechanism of steel wire in this paper. Then, the corrosion fatigue tests of smooth steel wire under different stress ratios and loading frequencies were carried out in different solutions. Through the comparative analysis of the corrosion fatigue life, side surface morphology, and fracture surface morphology of steel wire, the influence of corrosion environment and loading condition on the corrosion fatigue behavior of smooth steel wire was mainly discussed. 

## 2. Experimental

### 2.1. Long-Term Corrosion Test

Cold drawn pearlite steel wires (1 mm in diameter) collected from mine wire rope of 6 × 19 type were used as the test materials. The composition and mechanical properties of steel wire are shown in Table 1 and Table 2, respectively. The corrosion solutions used in the test were prepared according to the composition of spray water in different coalmines. The specific ionic components in the corrosion solutions are shown in Table 3.

In order to facilitate the lab research, full immersion method was used in the long-term corrosion test of steel wire. All specimens were embedded in epoxy resin with an exposure area of 1.0 cm^2^, and then they were placed in the dry box for 24 h. Steel wires were immersed in three different corrosion solutions, while 9 groups of parallel samples were used in each group. Each experiment under different conditions was conducted three times. The immersion period was 1 month. The solutions were replaced every three days. Measurements of polarization curves and electrochemical impedance spectroscopies were carried out on the electrochemical work station of IM6ex type. The test solution was in agreement with the immersion solution. The classic three electrode system was used in the long-term corrosion test, while the working electrode was used by wire samples, the reference electrode was used by Ag–AgCl electrode, and the auxiliary electrode was used by platinum wire. The polarization curves and electrochemical impedance measurements of steel wires soaking in different corrosion solutions were carried out at 1 h, 24 h, 96 h, 168 h, 240 h, 312 h, 432 h, 562 h and 720 h, respectively. When the polarization curves were measured, the potential scan range had an open-circuit potential (relative to the Ag–AgCl electrode, the same below) ±500 mV of specimen and a scan rate of 5 mV/s, while the electrochemical impedance spectroscopy (EIS) tests were carried out with a frequency of 10^5^–1 × 10^−2^ Hz and an AC signal amplitude of 5 mV [22,23]. Corrosion reaction kinetic parameters were analyzed by using Zsimwin software to fit the impedance data obtained in the experiment. The appropriate equivalent circuit was selected to analyze the surface state of steel wire [24]. 

In addition, the corrosion condition of the sample was evaluated by the average weight loss rate. The calculation method of the weight loss rate was as follows:

(1) $$v=\frac{\Delta w}{s\times t}$$

where Δ*w*, *s* and *t* are the weight loss, exposed area and immersion time of specimen, respectively.

### 2.2. Corrosion Fatigue Test

**Figure 1:**
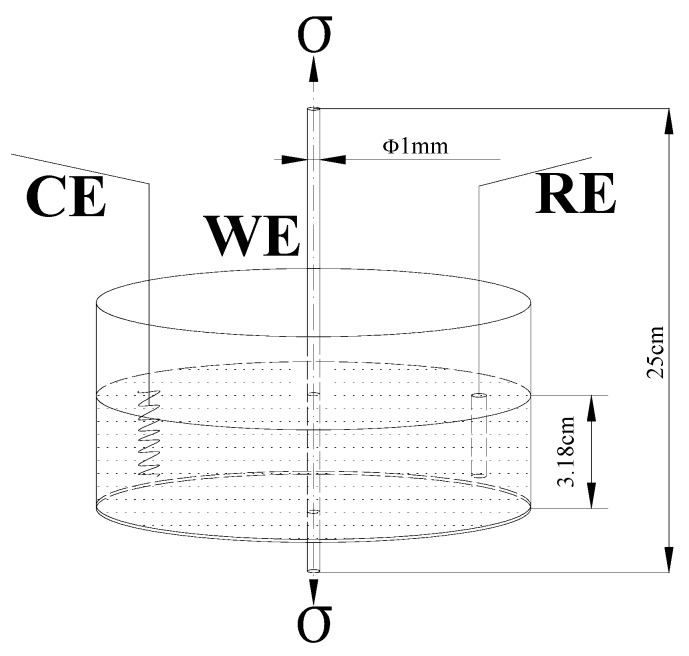
Schematic of corrosion fatigue test.

The corrosion fatigue (CF) test of steel wire was carried out on the hydraulic servo fatigue testing machine of PYW-20 type (Changchun Research Institute for Mechanical Science Co., Ltd., Changchun, China). The test principle is shown in Figure 1. The total length of steel wire was 25 cm, and the length of the sample soaking in solution was 3.18 cm, which ensured that the exposed area of steel wire in solution was 1 cm^2^. Firstly, the *S*-*N* (Stress-Number of Stress Cycles) curves and fracture morphologies of steel wires immersed both in air and three different corrosion solutions were compared under the frequency (*f*) of 10 Hz and the stress ratio (*r*) of 0.5, while the maximum applied stresses were 600 MPa, 700 MPa, 800 MPa, 900 MPa and 1000 MPa. Then, in order to study the formation and expansion mechanism of corrosion fatigue crack, the CF tests were carried out under different solutions, different *f* (2 Hz and 5 Hz) and different *r* (0.05, 0.25 and 0.5), while the maximum applied stress was 800 MPa. Constant control and sinusoidal loading were used in all CF tests. After the CF tests, surface and fracture morphologies were observed to discuss the CF mechanism of steel wire.

## 3. Results

### 3.1. Polarization Curves and Electrochemical Impedance Spectroscopy

**Figure 2:**
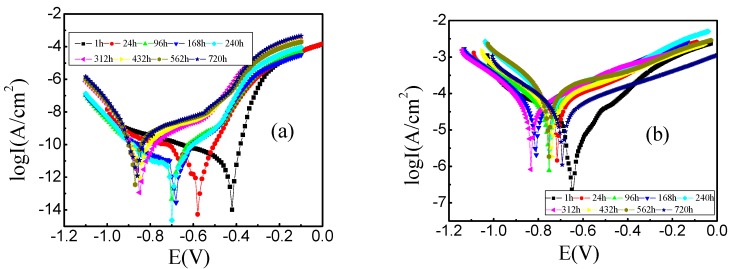

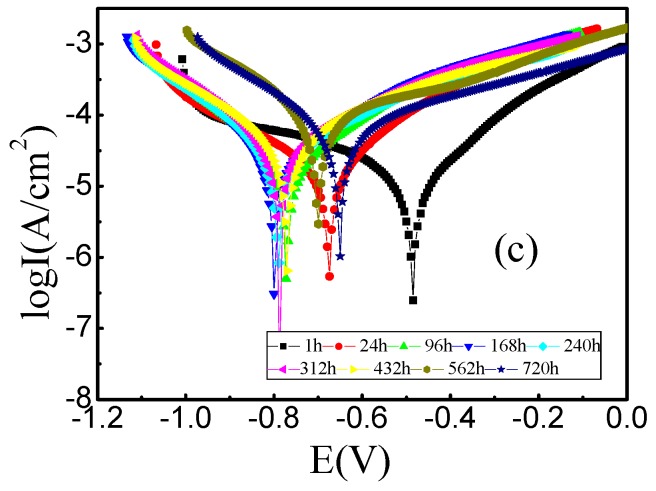
Polarization curves of mine steel wire in: (**a**) acid solution; (**b**) neutral solution; and (**c**) alkaline solution as the time extended.

The polarization curves of steel wires at different immersion times in three different corrosion solutions are shown in Figure 2. The anodic polarization curves of steel wires before 24 h in acid solution showed that the anodic reaction was a complete anodic dissolution process. After 24 h, the anodic dissolution process was inhibited under the protection of the surface corrosion product film. As shown in Figure 2b,c, after 1 h in neutral and alkaline solution, the anode corrosion current density increased slowly with the increasing of corrosion potential, which showed that the corrosion product film had a certain protection on the substrate and hindered the process of anodic dissolution. The corrosion product film formed on the surface of steel wire after 1 h in acid solution was quickly decomposed. The corrosion product film formed in neutral and alkaline solution could not be quickly decomposed, which had a protective effect on the substrate. With the extension of the immersion time, there was always a similar platform in anode curve of steel wire in all three kinds of solutions. The anode curve slopes were similar both in neutral and alkaline solution, while in acid solution, the corrosion current density increased rapidly with the further expanding of corrosion potential, which was mainly due to that further anodic dissolution reaction occurred under the high anodic potential for the higher corrosivity of acid solution.

**Figure 3:**
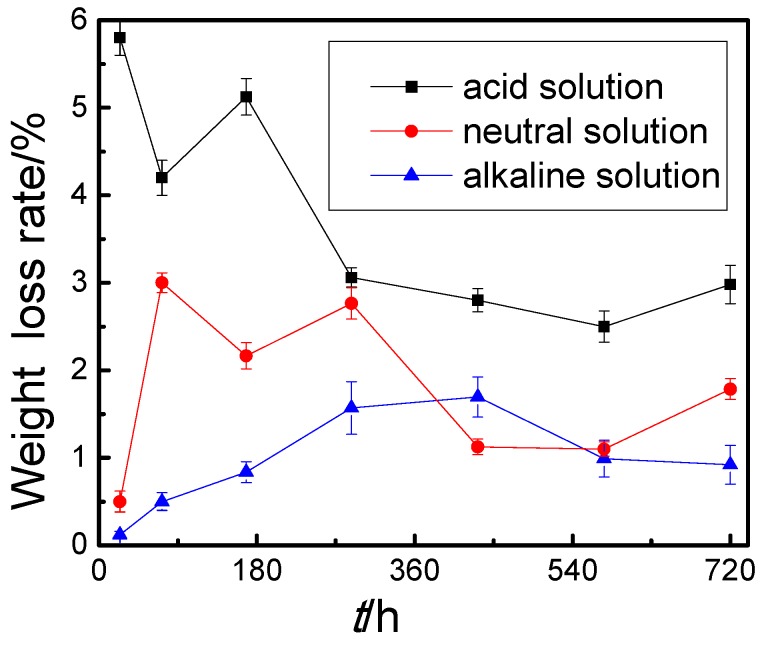
Weight loss ratios of steel wires in three solutions at different immersion times.

Figure 3 shows the weight loss ratios of steel wires in three solutions at different immersion times. The weight loss rate of steel wire gradually decreased with the extension of immersion time in acid solution, which reached 0.058 mg·(cm^2^·h)^−1^ after 24 h and then decreased rapidly. It reached 0.058 mg·(cm^2^·h)^−1^ after soaking for 288 h, and gradually stabilized during 288–720 h. In neutral solution, the weight loss rate of steel wire was only 0.005 mg·(cm^2^·h)^−1^ after soaking for 24 h, and rapidly increased to 0.03 mg·(cm^2^·h)^−1^ at 72 h. After 288 h, the weight loss rate reduced to about 0.01 mg·(cm^2^·h)^−1^ and kept stable. In alkaline solution, the weight loss rate was close to zero in early immersion time, and slowly increased with the extension of immersion time. After soaking for 432 h, the weight loss rate increased to 0.017 mg·(cm^2^·h)^−1^, then slowly decreased and stabilized at 0.01 mg·(cm^2^·h)^−1^.

**Figure 4:**
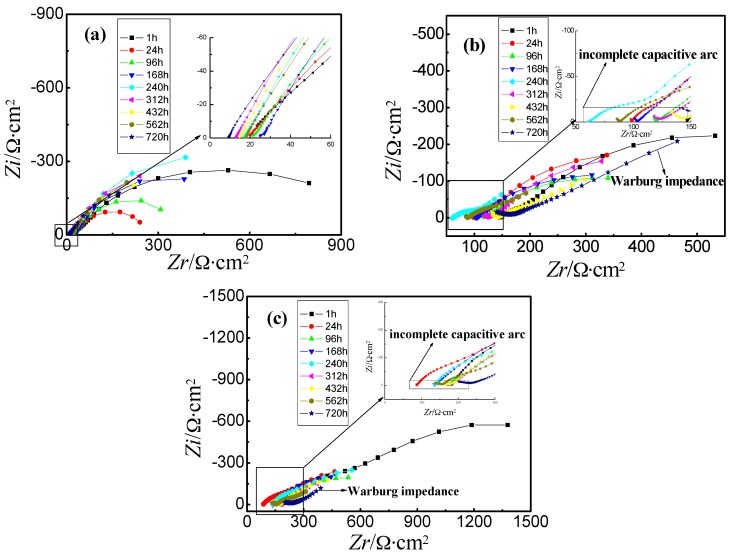
Nyquist plots of untreated steel wire in three different solutions at different immersion times.

Figure 4 shows the Nyquist diagram of steel wire immersed in three different corrosion solutions after soaking for different times. At different immersion times, the resistance of acid solution was the smallest, while the resistance of alkaline solution was the biggest. With the extension of immersion time, there was an incomplete capacitive arc in high frequency region of Nyquist diagram in neutral and alkaline solution, which was because a layer of rust could be generated on the surface of steel wire. The rust would not exist in acid solution due to the high corrosivity.

### 3.2. S-N Curve

**Figure 5:**
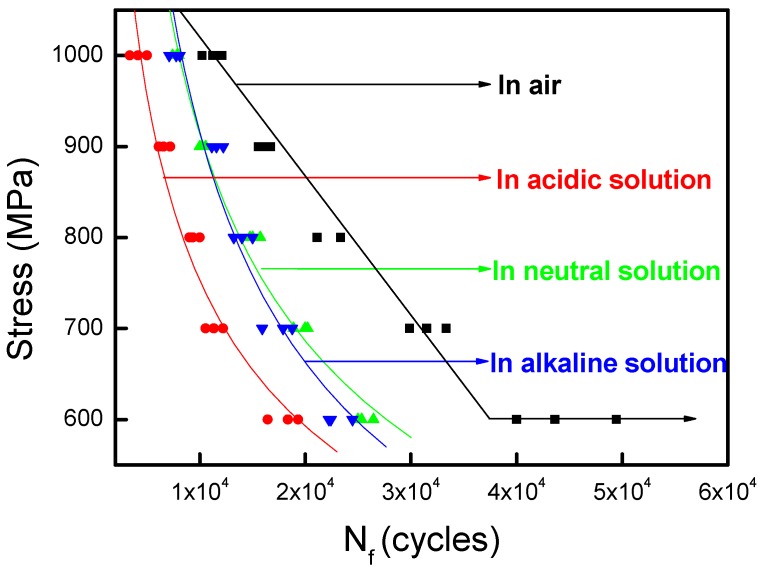
*S-N* curves of steel wire under different environments.

The *S-N* curves of mine steel wire in air and three different corrosion solutions are given in Figure 5. The maximum applied stress was 600 MPa, 700 MPa, 800 MPa, 900 MPa and 1000 MPa, the stress ratio was 0.5, and the frequency was 10 Hz. The corrosion fatigue behaviors of steel wires in different mediums were significantly different. With the increase of the maximum stress, the fatigue life in air increased faster than that in solutions. The corrosion fatigue life of steel wire in acid solution was the lowest for the highest corrosivity, and which in neutral and alkaline solution were not very different. By fitting the experimental data, the relationships of corrosion fatigue life of steel wire in three solutions were shown as follows [25]:

Acid solution: lg*S* = 4.2617 − 0.34622 × lg*N_f_*;
Neutral solution: lg*S* = 4.61641 − 0.41385 × lg*N_f_*; and
Alkaline solution: lg*S* = 4.82836 − 0.4666 × lg*N_f_*.

In the above, *S* and *N_f_* are the applied stress and the number of cycles, respectively.

**Figure 6:**
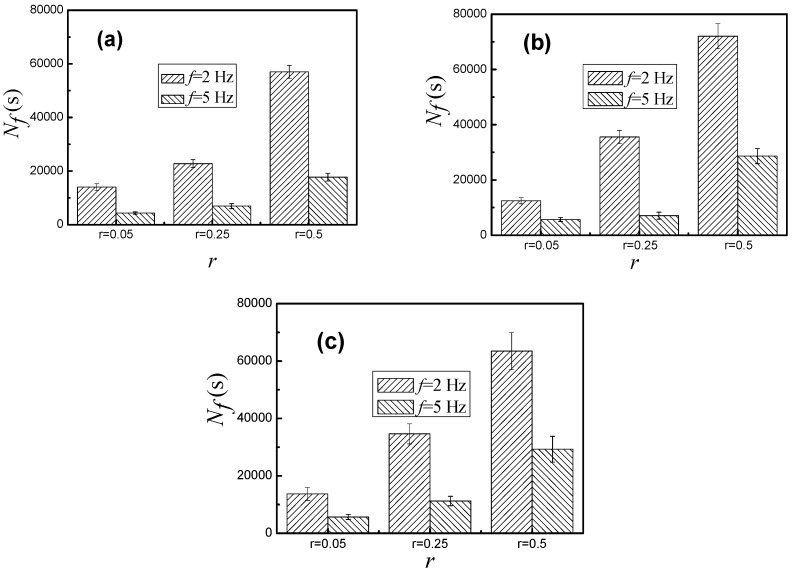
Corrosion fatigue life of steel wire under different stress ratios and frequencies in: (**a**) acid solution; (**b**) neutral solution; and (**c**) alkaline solution.

Figure 6 showed the corrosion fatigue of steel wire in three different corrosion solutions under different stress ratios and frequencies, while the maximum stress was 800 MPa. The effect of stress ratio and frequency on the corrosion fatigue life of steel wire was very great. The corrosion fatigue life of steel wire increased with the increase of stress ratio under the same frequency, and decreased with the increase of frequency under the same stress ratio. When the loading frequency was 2 Hz, the corrosion fatigue life of steel wire increased with the increase of stress ratio. The speed of increasing was much faster than that under the frequency of 5 Hz. No matter what kind of solutions, the corrosion fatigue life of steel wire was the lowest under the stress ratio of 0.05 and the frequency of 5 Hz, which was the highest under the stress ratio of 0.5 and the frequency of 2 Hz. In neutral solution, the highest corrosion fatigue life of steel wire was about 72,000 cycles under the frequency of 2 Hz and the stress ratio of 0.5. The lowest life was about 5700 cycles under the frequency of 5 Hz and the stress ratio of 0.05. The biggest difference on the corrosion fatigue life of steel wire under different loading conditions in neutral solution was about 66,300 cycles, which in acid solution was about 52,700 cycles. The results showed that the effect of stress ratio and frequency on the corrosion fatigue life of steel wire was influenced by the corrosion resistance of the solution: the stronger the corrosion resistance of the solution, the smaller the effect. In addition, the corrosion fatigue life of steel wire under various conditions was the lowest in acid solution. The difference on the corrosion fatigue life in the neutral solution and alkaline solution was very small.

### 3.3. Morphology on Side Surface and Fracture Surface of Steel Wire

**Figure 7:**
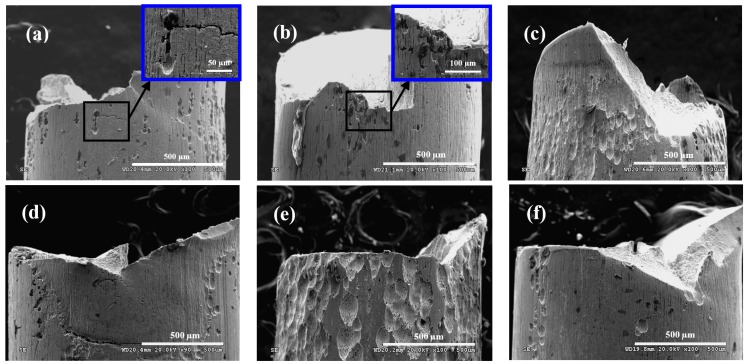
Morphologies on side surface of steel wire under: (**a**) *r* = 0.05, *f* = 2 Hz; (**b**) *r* = 0.05, *f* = 5 Hz; (**c**) *r* = 0.25, *f* = 2 Hz; (**d**) *r* = 0.25, *f* = 5 Hz; (**e**) *r* = 0.5, *f* = 2 Hz; and (**f**) *r* = 0.5, *f* = 5 Hz in acid solution.

Figure 7 shows the morphologies on side surface of steel wire under different loading conditions in acid solution. Under the frequency of 2 Hz (Figure 7a,c,e), the corrosion on the surface of steel wire was serious, which was aggravated with the increasing of stress ratio. When the stress ratio was 0.05 (Figure 7a), there were obvious cracks in the direction perpendicular to the applied stress, which also had a tendency to extend along the tensile direction. When the stress ratio was 0.5 (Figure 7e), there were many corrosion pits covered on the surface of steel wire, which might have covered the crack. Under the frequency of 5 Hz (Figure 7b,d,f), the corrosion on the surface of steel wire were slight. The change of stress ratio has little influence on the morphology on side surface of steel wire. When the stress ratio was 0.05 (Figure 7b), unobvious crack could be found near the fracture of steel wire, and, when the stress ratio was 0.5 (Figure 7f), there was no crack on the side surface of steel wire because the corrosion pits were very deep.

**Figure 8:**
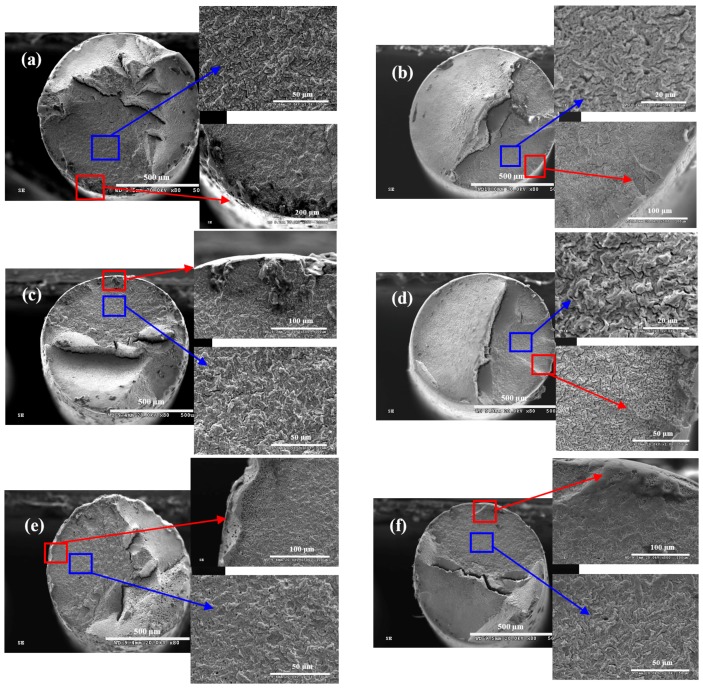
Wire fracture morphologies under: (**a**) *r* = 0.05, *f* = 2 Hz; (**b**) *r* = 0.05, *f* = 5 Hz; (**c**) *r* = 0.25, *f* = 2 Hz; (**d**) *r* = 0.25, *f* = 5 Hz; (**e**) *r* = 0.5, *f* = 2 Hz; and (**f**) *r* = 0.5, *f* = 5 Hz in acid solution.

Figure 8 shows the morphologies on fracture surface of steel wire under different loading conditions in acid solution, which also showed the morphologies of fatigue crack source (red box) and crack propagation zone (blue box). In acid solution, the morphologies on fracture surfaces of steel wires showed that the specimens had multi-fatigue crack source. The fracture surface under the stress ratio of 0.05 and the frequency of 5 Hz was the smoothest, while the fracture surface under the stress ratio of 0.5 and the frequency of 2 Hz was the most rough. In addition to the condition that the stress ratio was 5 Hz and the frequency was 0.05, dimple morphology could be found on the fatigue crack source of steel wire under the five other conditions, which was the most obvious under the stress ratio of 0.5 and the frequency of 2 Hz. As shown in Figure 8b, the fatigue crack source was worn under the stress ratio of 0.05 and the frequency of 5 Hz, which is because the upper and lower surfaces of steel wire in the cracked place collided with each other under the fast crack propagation rate and low stress ratio [26]. The original fracture surface of the fatigue crack source was destroyed by the common reaction of low stress ratio and high loading frequency. In addition, a large number of tiny corrosion pits could be found on the fatigue crack propagation zone, which was due to the strong corrosivity of the acid solution.

**Figure 9:**
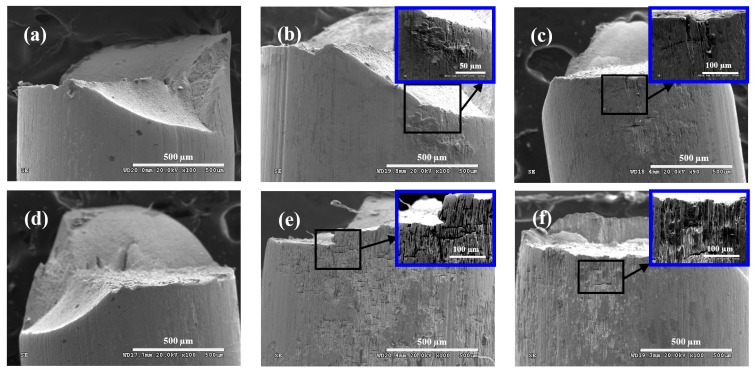
Morphologies on side surface of steel wire under: (**a**) *r* = 0.05, *f* = 2 Hz; (**b**) *r* = 0.05, *f* = 5 Hz; (**c**) *r* = 0.25, *f* = 2 Hz; (**d**) *r* = 0.25, *f* = 5 Hz; (**e**) *r* = 0.5, *f* = 2 Hz; and (**f**) *r* = 0.5, *f* = 5 Hz in neutral solution.

Figure 9 shows the morphologies on side surface of steel wire under different loading conditions in neutral solution. Regardless of the loading conditions, there were no obvious corrosion pits on the side surface of steel wire. In addition, under the same frequency, the cracks on the side surface of steel wire could be found gradually with the increasing of stress ratio. In particular, Figure 9e,f shows that a large number of long and thin cracks with a tendency to extend along the tensile direction gathered near the fatigue crack source of steel wire. Meanwhile, the change of frequency had little influence on the side surface morphologies of steel wires.

**Figure 10:**
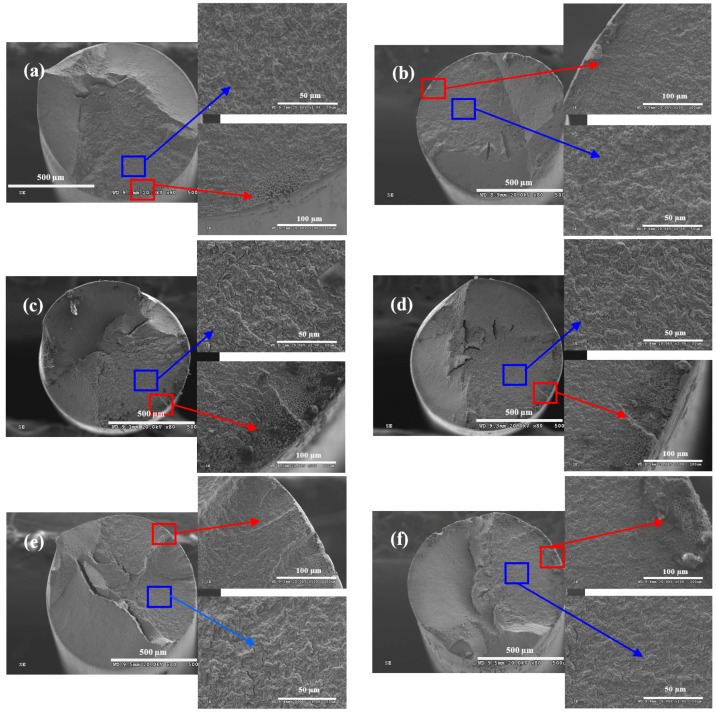
Wire fracture morphologies under: (**a**) *r* = 0.05, *f* = 2 Hz; (**b**) *r* = 0.05, *f* = 5 Hz; (**c**) *r* = 0.25, *f* = 2 Hz; (**d**) *r* = 0.25, *f* = 5 Hz; (**e**) *r* = 0.5, *f* = 2 Hz; and (**f**) *r* = 0.5, *f* = 5 Hz in neutral solution.

Figure 10 shows the morphologies on fracture surface of steel wire under different loading conditions in neutral solution. The fracture surface of steel wire in neutral solution was much smoother than that in acid solution. There was only one fatigue crack source on the fracture surface when *r* was 0.05, while there were multi-fatigue crack sources on the fracture surface when *r* was 0.5 and *f* was 2 Hz. There were no visible dimples in the region of fatigue crack source and obvious quasi cleavage fracture in the crack propagation zone when *r* was 0.05 and *f* was 5 Hz. There were some well-distributed corrosion pits on the quasi cleavage plane. This morphology indicated that the crack formation of steel wire was mainly controlled by the deformation activation mechanism. The anodic dissolution mechanism was less affected under this condition. Under the five other conditions, there were visible dimples in the region of fatigue crack source, which indicated that the crack formation was mainly controlled by the anodic dissolution process. The morphologies of crack propagation zones mainly showed quasi cleavage fracture. The transgranular fracture occurred along the crystallographic plane. When *r* was 0.5 and *f* was 2 Hz, there were secondary cracks in the crack propagation zone of steel wire, which were perpendicular to the direction of the main crack propagation.

**Figure 11:**
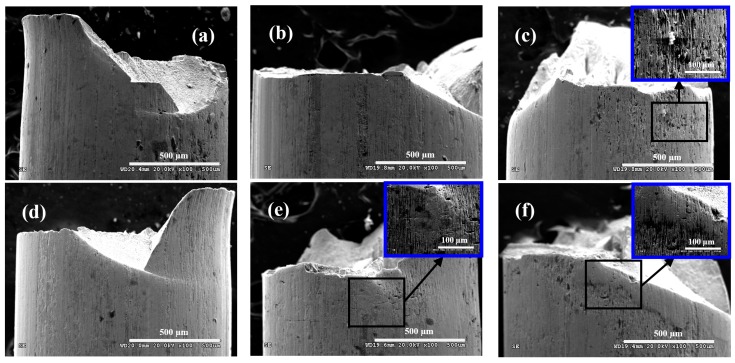
Morphologies on side surface of steel wire under: (**a**) *r* = 0.05, *f* = 2 Hz; (**b**) *r* = 0.05, *f* = 5 Hz; (**c**) *r* = 0.25, *f* = 2 Hz; (**d**) *r* = 0.25, *f* = 5 Hz; (**e**) *r* = 0.5, *f* = 2 Hz; and (**f**) *r* = 0.5, *f* = 5 Hz in alkaline solution.

Figure 11 shows the morphologies on side surface of steel wire under different loading conditions in alkaline solution. The morphologies on the surface of steel wires in neutral solution and alkaline solution were similar.

**Figure 12:**
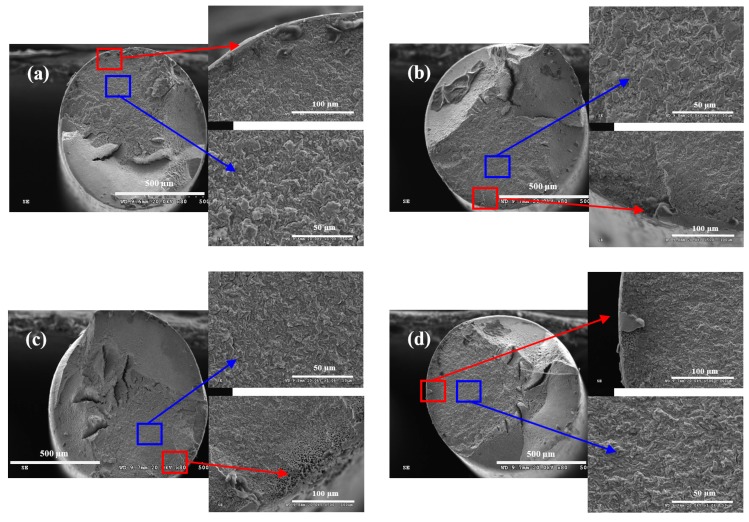

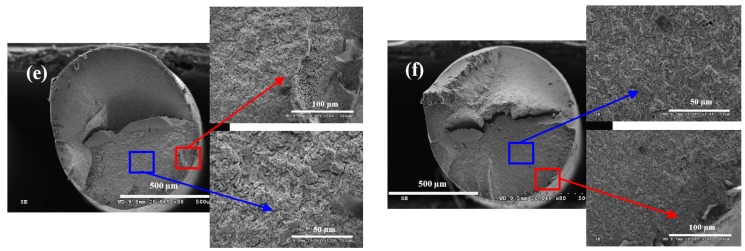
Wire fracture morphologies under: (**a**) *r* = 0.05, *f* = 2 Hz; (**b**) *r* = 0.05, *f* = 5 Hz; (**c**) *r* = 0.25, *f* = 2 Hz; (**d**) *r* = 0.25, *f* = 5 Hz; (**e**) *r* = 0.5, *f* = 2 Hz; and (**f**) *r* = 0.5, *f* = 5 Hz in alkaline solution.

Figure 12 shows the morphologies on fracture surface of steel wire under different loading conditions in alkaline solution. Under the conditions of *r* = 0.05 and *f* = 5 Hz, *r* = 0.05 and *f* = 2 Hz, and *r* = 0.25 and *f* = 5 Hz, there was one fatigue crack source with less dimples on the fracture surface. Similar to neutral solution, the fatigue crack source and crack propagation zone were seriously worn when *r* was 0.05 and *f* was 5 Hz in alkaline solution, which also verified that the lower the stress ratio, the slower the crack propagation. Under the five other conditions, the crack propagation zone mainly showed quasi cleavage fracture, which indicated that the crack propagation of steel wire followed the brittle fracture mechanism.

## 4. Discussion

### 4.1. The Electrochemistry of Corrosion

As shown in Figure 2, after soaking for 240 h in acid solution, the corrosion current of steel wire decreased with the increasing of potential, but was not sharply reduced in specific potential, which might be due to that the cathodic reaction of steel wire gradually changed from reductive of hydrogen ions to reductive of oxygen with the extension of immersion time. Conversely, in neutral and alkaline solutions, the cathodic reaction of steel wire has been the reduction of hydrogen ion during the whole soaking process. The different cathodic reactions are shown, respectively, as follows [27]:

Reduction of hydrogen ion: 2*H^+^* + 2*e* = *H*_2_*↑*

Reduction of oxygen: *O*_2_ + 4*H^+^* + 4*e* = 2*H*_2_*O*

The formation of corrosion product film was demonstrated by the inconspicuous platform in the anodic polarization curve, which hindered the anodic dissolution process of steel wire. The corrosion product film was penetrated by ion with the increase of polarization potential in acid solution, while pitting corrosion occurred on local surface of steel wire. This phenomenon could not be easy to be found in neutral and alkaline solution. In the late immersion, the protection of corrosion product film on the matrix was greatly weakened for the long-term erosion. The system tended to reach stability state when the formation and damage rate of corrosion product film tended to be consistent [28].

The weight loss rate of steel wire was bigger during the initial immersion in acid solution, which was because the corrosion product was also dissolved for the strong corrosivity of acid solution. In medium-term and late immersion, the weight loss rates tended to be stable for the protective effect of corrosion product film on the matrix of steel wire. In neutral and alkaline solutions, the weight loss rate in initial immersion was smaller for the weak corrosivity of solutions and the protective effect of phosphate film [29]. With the extension of immersion time, corrosion products were formed on the surface of steel wire for the loss of phosphate coating. In addition, the OH^−^ of alkaline solution had a certain impediment to the corrosion of steel wire [30,31]. The difference of weight loss rate reflected the effect of different solutions on the corrosion behavior of steel wire. With the extension of immersion time, the differences of weight loss rates were smaller and smaller, which reflected that the corrosion product film formed during the immersion process had a certain degree of protection to the steel wire. Although the corrosion characteristics of steel wire in different solutions were different, the influence of different solutions on the corrosion behavior of steel wire was getting smaller and smaller with the extension of immersion time. 

**Figure 13:**
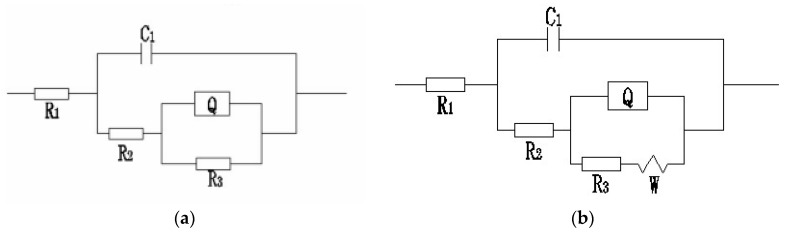
Equivalent circuits obtained by Nyquist plots. (**a**) *R(C(R(QR)))*; (**b**) *R(C(R(Q(RW))))*.

According to the results of EIS tests, the resistance of electrolyte solution had not changed obviously, which ensured the reliability of the experiment. The Nyquist diagrams simulated by Zsimwin software were used to get the equivalent circuits of steel wires in different solutions, which are shown in Figure 13. Among them, *R*_1_ represented the resistance of corrosion solution, *C*_1_ and *R*_2_ represented the capacitors and resistors of corrosion product film, respectively, *C*_2_ of CPE element parameter represented the double layer capacitance of matrix and corrosion product film, *n* represented the diffusion coefficient of non ideal capacitor, and *R_3_* represented the electric double layer charge transfer resistance [22,23]. Table 4, Table 5 and Table 6 show the corrosion reaction kinetic parameters of steel wires in different solutions, and Table 7 shows the equivalent circuit diagrams under different conditions. There was little difference in the kinetic parameters of corrosion reaction of steel wire immersed in neutral solution and alkaline solution. Diffusion tail appeared in the low frequency region after soaking for 720 h in neutral and alkaline solutions, which was the characteristic of typical Warburg impedance [32]. At this immersion time, the corrosion process of steel wire was controlled by the diffusion of O or Fe ion. In acid solution, the capacitance of corrosion product film was bigger than that in the two other solutions. Although the resistance in acid solution was not very different from which in the other two solutions, the transfer resistance of the double layer was smaller. This was because the acid solution with strong corrosivity reduced the protective effect of corrosion product film on the matrix. This result was also consistent with the results of polarization curves and weight loss rate curves.

### 4.2. Corrosion Fatigue Mechanism

Because of the strong corrosivity of acid solution, the corrosion pits formed on the surface of steel wire became the corrosion fatigue crack source under the loading stresses. The initiation and propagation rate of crack increased significantly with the increase of the applied stresses, which were the fastest in acid solution. According to the study of Ford [33], galvanic cell was formed between the deformation zone (anode region) and the non deformed region of metal (cathode region), while the corrosion fatigue crack source was formed by the dissolution of anode region. The area of anode region would become larger under the large loading stress, which caused that the dissolution rate of anode region became faster. The corrosion fatigue life of metal materials was mainly related to the formation period and the extension period of corrosion fatigue crack, which could be expressed as follows [34]:

*N_f_* = *N_i_* + *N_p_* (2)

where *N_f_*, *N_i_* and *N_p_* are the corrosion fatigue life, the formation period life and the extension period life of corrosion fatigue crack, respectively.

For pre-crack metal specimen, the corrosion fatigue life was mainly controlled by the crack propagation rate of crack. For smooth steel wire, the formation period life of crack played an important part on the corrosion fatigue life of steel wire. For the crack initiation mechanism of anodic dissolution, when the corrosivity of solution was much stronger, the corrosion pits would be more easily to be formed on the surface of steel wire. While for the crack initiation mechanism of deformation activation, the lower stress ratio and higher frequency could cause the greater work of deformation activation energy, which would accelerate the generation of corrosion fatigue crack source. This was contrary to the results of pre-cracked specimens. In Han’s study [19], pH at crack tip decreased with crack growing: the lower the stress ratio and frequency were, the lower the pH in local solution was. 

According to the morphology of side surface, the morphological properties on the side surface of steel wire under different conditions are summarized in Table 8. It was considered that the formation of crack was related to the size of stress ratio in acid solution: the lower the stress ratio was, the more easily the crack appearance was observed, while the higher the stress ratio was, the more serious the corrosion on the side surface of steel wire was. There was also no obvious crack in acid solution, which might be due to that the cracks were covered by the corrosion pits. Under the same stress ratio, the lower the frequency was, the more serious the corrosion on the side surface of steel wire was. Under different stress ratios and frequencies in acid solution, the stress concentration was formed by the corrosion pits, which would be the source of fatigue crack. The side surface of steel wire mainly showed crack morphology with less corrosion pits in neutral solution and alkaline solution. The higher the stress ratio was, the more easily the transverse crack could be found.

According to the fracture and side surface morphologies of steel wire, the initiation mechanism of corrosion fatigue crack under different conditions are summarized in Table 9. When *r* was 0.05 and *f* was 5 Hz in acid solution, the formation of corrosion fatigue crack was mainly controlled by the deformation activation and anodic dissolution mechanism, while the nucleation of cracks was accelerated by the strong corrosivity of acid solution. Under the five other conditions in acid solution, the corrosion fatigue crack propagation zone of steel wire mainly presented the brittle fracture morphology. In the process of fatigue crack propagation, local plastic deformation occurred on the intergranular fracture surface. The significant plastic deformation occurred at every load cycle. Finally, a classic fatigue strip was formed on the fracture surface. By comparison, it was found that in acid solution, the higher the loading frequency and the lower the stress ratio, the more smooth the side surface of steel wire and the more obvious the dimple morphology in fatigue crack propagation zone. The lower the loading frequency and the higher the stress ratio, the rougher the side surface of steel wire and the more obvious the quasi cleavage fracture morphology in fatigue crack propagation zone.

Due to the weak corrosivity of neutral solution, the corrosion pits could not be formed on the side surface of steel wire in short time, which indicated that the crack formation of steel wire was mainly affected by the deformation activation when *r* was 0.05 and *f* was 5 Hz. The speed of fatigue crack propagation was slow under small stress ratio. With the propagation of the fatigue crack, a strong acid corrosion environment was generated at crack tip, which caused the formation of corrosion pits in the crack propagation zone. The number of corrosion pits in the crack propagation zone in neutral solution was much less than that in acid solution, in addition, due to the inhibitory effect of OH^−^ on the anodic dissolution process in alkaline solution, there was no corrosion pits in the crack propagation zone when *r* was 0.05 and *f* was 5 Hz. Under the five other conditions, morphologies of corrosion fatigue crack source in acid and neutral solution showed that the crack initiation was mainly controlled by anodic dissolution mechanism. The hydrogen evolution process of cathodic reaction also played an important role in the initiation and propagation of crack, while in alkaline solution, the cathodic hydrogen evolution reaction was hindered by OH^−^, which caused that the crack initiation could not be nucleation in short time. At this time, the crack initiation was mainly related to steel wire itself defects and deformation activation [4,5,6]. Although the anodic dissolution mechanism played a certain role, the effect was weaker.

## 5. Conclusions

In summary, we first studied the electrochemical corrosion behavior of smooth steel wire in coalmine. The results of polarization curves and weight loss curves showed that the corrosion of steel wire was the most severe in acid solution. At late stage of immersion, the cathodic reaction of steel wire gradually changed from the reduction of hydrogen ion to the reduction of oxygen in acid solution, whereas this was always the reduction of hydrogen ion in neutral and alkaline solutions. The Nyquist diagrams simulated by Zsimwin software were used to get the equivalent circuits of steel wires. The results showed that the equivalent circuits of steel wires in acid solutions would always be *R(C(R(QR)))* at different immersion times. The appearance of Warburg impedance in Nyquist plots showed that the equivalent circuits in neutral and alkaline solutions would be *R(C(R(Q(RW))))* at late stage of immersion time. The effect of stress ratio and loading frequency on the corrosion fatigue mechanism of steel wire in different solutions was also discussed in this paper. The effect on the crack initiation mechanism was emphasized. The strong corrosivity of acid solution could accelerate the nucleation of the crack tip. The initiation mechanism of corrosion fatigue crack under different conditions was summarized according to the side surface and fracture surface morphologies. Of course, the effect of stress ratio and loading frequency on the crack propagation progress of smooth steel wire was hard to study, and still needs further experimental verification.

## Figures and Tables

**Table 1:** Chemical composition of steel wire (in wt. %).

Composition	Fe	Mn	Si	Ni	C	S	P
Percentage	94.62	4.53	0.02	0.01	0.84	0.001	<0.001

**Table 2:** Mechanical properties of steel wire.

Mechanical Properties	Tensile Strength	Yield Strength	Young Modulus	Elongation Ratio	Section Shrinkage
Value	1750 MPa	1300 MPa	210 GPa	24.19%	34.78%

**Table 3:** Typical water quality composition of coalmine.

pH	Ion Content/mg/L								
	H^+^	K^+^	Na^+^	Ca^2+^	Mg^2+^	Cl^−^	SO_4_^2−^	OH^−^	HCO_3_^−^
2.97	1.28	11.73	92.42	676.55	364.74	28.36	3283.81		
6.97		11.73	141.16	80.56	49.33	89.33	551.38		
9.97		11.73	35.63	54.43	36.45	89.33	232.95	3.33	18.92

**Table 4:** Corrosion kinetic parameters of steel wires in acid solution.

Time	*R*_1_	*C*_1_	*R*_2_	*Q*		*R*_3_
h	Ω·cm^2^	F·cm^−2^	Ω·cm^2^	C_2_/F·cm^−2^	n	Ω·cm^2^
1	21.25	2.012 × 10^−5^	21.8	4.993 × 10^−4^	0.6385	952.8
24	19.97	1.43 × 10^−4^	10.09	1.397 × 10^−3^	0.7225	260.8
96	21.03	1.213 × 10^−4^	4.484	1.698 × 10^−3^	0.7212	411.1
168	24.78	1.394 × 10^−4^	4.189	1.908 × 10^−3^	0.7383	719.8
240	16.89	2.537 × 10^−4^	5.005	1.932 × 10^−3^	0.7171	1051
312	12.89	5.191 × 10^−4^	4.404	3.063 × 10^−3^	0.7176	887.3
432	14.08	7.231 × 10^−5^	1.063	3.814 × 10^−3^	0.7472	712.7
562	17.75	7.686 × 10^−5^	1.299	4.315 × 10^−3^	0.7289	990
720	9.023	1.643 × 10^−4^	0.306	4.783 × 10^−3^	0.7136	717.8

**Table 5:** Corrosion kinetic parameters of steel wires in neutral solution.

Time	*R*_1_	*C*_1_	*R*_2_	*Q*		*R*_3_	*W*
h	Ω·cm^2^	F·cm^−2^	Ω·cm^2^	C_2_/F·cm^−2^	n	Ω·cm^2^	
1	147.4	5.386 × 10^−5^	29.38	2.013 × 10^−3^	0.6414	854.1	
24	99	6.955 × 10^−5^	24.12	1.878 × 10^−3^	0.6708	632.9	
96	110.6	1.407 × 10^−6^	9.886	3.065 × 10^−3^	0.5822	470.1	
168	97.64	2.968 × 10^−7^	5.959	3.394 × 10^−3^	0.5783	504	
240	60.96	1.5 × 10^−5^	3.075	1.373 × 10^−3^	0.7108	39.51	1.472 × 10^−2^
312	114.1	3.37 × 10^−7^	9.592	4.666 × 10^−3^	0.5183	1905	
432	119	6.962 × 10^−7^	20.37	6.12 × 10^−3^	0.3636	1713	
562	81.4	2.378 × 10^−7^	6.553	3.516 × 10^−3^	0.5668	101.5	1.249 × 10^−2^
720	123	4.683 × 10^−8^	23.19	2.103 × 10^−3^	0.3135	98.1	1.097 × 10^−3^

**Table 6:** Corrosion kinetic parameters of steel wires in alkaline solution.

Time	*R*_1_	*C*_1_	*R*_2_	*Q*		*R*_3_	*W*
h	Ω·cm^2^	F·cm^−2^	Ω·cm^2^	C_2_/F·cm^−2^	n	Ω·cm^2^	
1	183.9	1.341 × 10^−4^	547.2	0.0008045	0.6958	1764	
24	87.81	2.89 × 10^−8^	242.8	0.001643	0.7307	679.8	
96	136.9	1.21 × 10^−8^	34.69	0.002154	0.7212	994.7	
168	122.5	7.41 × 10^−8^	13.53	0.002572	0.5383	931.1	
240	137.6	4.32 × 10^−8^	5.005	0.001932	0.7171	536.8	
312	148.6	5.251 × 10^−8^	23.79	0.005063	0.5176	651.1	
432	157.1	3.231 × 10^−7^	17.15	0.003478	0.5472	1439	
562	127.5	9.686 × 10^−8^	5.509	0.003954	0.4289	39.21	1.704 × 10^−3^
720	189.6	7.537 × 10^−8^	18.5	0.002326	0.5136	98.73	3.888 × 10^−3^

**Table 7:** Equivalent circuits of steel wires in different solutions at different immersion times.

Solution	Initial Immersion (1 h)	Medium-Term Immersion (24–240 h)	Late Immersion (312–720 h)
Acid solution	*R(C(R(QR)))*	*R(C(R(QR)))*	*R(C(R(QR)))*
Neutral solution	*R(C(R(QR)))*	*R(C(R(QR)))*	*R(C(R(Q(RW))))*
Alkaline solution	*R(C(R(QR))))*	*R(C(R(QR)))*	*R(C(R(Q(RW))))*

**Table 8:** Morphological property on the side surface of steel wire.

Conditions	Acid Solution	Neutral Solution	Alkaline Solution
*r* = 0.05, *f* = 2 Hz	P/C	None	None
*r* = 0.05, *f* = 5 Hz	Unobvious	None	None
*r* = 0.25, *f* = 2 Hz	P	C	C
*r* = 0.25, *f* = 5 Hz	P/C	None	None
*r* = 0.5, *f* = 2 Hz	P	C	C
*r* = 0.5, *f* = 5 Hz	P	C	C

C: crack; P: pit.

**Table 9:** Initiation mechanism of corrosion fatigue crack under different conditions.

Conditions	Acid Solution	Neutral Solution	Alkaline Solution
*r* = 0.05, *f* = 2 Hz	A	A	D
*r* = 0.05, *f* = 5 Hz	A/D	D	D
*r* = 0.25, *f* = 2 Hz	A/D	A	A
*r* = 0.25, *f* = 5 Hz	A	A	D
*r* = 0.5, *f* = 2 Hz	A	A/D	A
*r* = 0.5, *f* = 5 Hz	A	A	D

A: anodic dissolution mechanism; D: deformation activation mechanism.
